# The Impact of a Lacto-Ovo Vegetarian Diet on Post-Operative Recovery and Wound Healing Following Mandibular Third Molar Extraction—A Prospective Study

**DOI:** 10.3390/nu17050759

**Published:** 2025-02-21

**Authors:** Alicja Baranowska, Artur Pitułaj, Michał Makar, Konrad Kowalewski, Sebastian Dominiak, Paweł Kubasiewicz-Ross

**Affiliations:** 1 Dental Teaching Hospital, Wroclaw Medical University, Krakowska 26 St., 50-425 Wroclaw, Poland; alicja.baranowska99@gmail.com (A.B.); m.makar@op.pl (M.M.); kowalewskistomatolog@gmail.com (K.K.); 2 Oral Surgery Department, Wroclaw Medical University, Krakowska 26 St., 50-425 Wroclaw, Poland; artur.pitulaj@umw.edu.pl (A.P.); sebastian.dominiak95@wp.pl (S.D.)

**Keywords:** wound healing, vegetarian diet, third molar surgery, soft tissue healing

## Abstract

**Background/Objectives**: Despite the increasing popularity of lacto-ovo vegetarian (LOV) diets, their impact on oral wound healing remains underexplored. The aim of this study was to evaluate the potential influence of the LOV diet on soft tissue healing following lower wisdom tooth operation. **Methods**: This prospective study involved 40 participants equally divided into two groups: the LOV group (lacto-ovo vegetarians for at least seven years) and the control group (general diet). The main inclusion criterion was the need for third molar extraction, while smoking, pregnancy, and systemic diseases that may compromise wound healing were disqualifying factors. Parameters such as wound length, swelling, pain, analgesic use, and bacterial plaque index were assessed on the 3rd and 7th days post-surgery. **Results:** LOV participants exhibited significantly faster wound healing, with reduced wound length and swelling by the 7th day compared to the control group. Pain levels and analgesic consumption were also lower in the LOV group at the end of the observation period. Although the LOV group had higher trismus on the 3rd day, it normalized by the 7th day. These outcomes may be, apart of the diet type followed, partially attributed to the younger age and shorter surgical time observed in the LOV group. **Conclusions:** The findings highlight the potential role of dietary interventions in optimizing recovery, warranting further research to confirm these benefits in broader populations.

## 1. Introduction

Wound healing is a highly energy-intensive process that requires an adequate intake of macronutrients (proteins, carbohydrates, fats), fluids, and micronutrients such as vitamins and minerals [1,2,3]. The positive influence of proper nutrition on post-operative healing has been documented in numerous studies. Most of the studies emphasize the importance and positive correlation of a balanced diet with post-operative wound healing. Reports indicate that malnutrition or undernutrition frequently leads to delayed wound healing, often complicated by wound infections, secondary healing, or even failure to heal [1,2,3].

Plant-based diets are a unique group of restrictive diets characterized by the limitation or complete exclusion of meat-based dishes, rooted in both ethical and health considerations [4].

Despite the clear definition of such dietary categories, there is a wide variety of dietary patterns. Vegetarians can be divided into subgroups: semi-vegetarians, defined as those consuming red meat and poultry once per month or more, and all other meats—including fish—once per month or more, but no more than once per week; pesco-vegetarians, who consume fish once per month or more but all other meats less than once per month; lacto-ovo vegetarians, who consume eggs and dairy once per month or more but fish and other meats less than once per month; and finally, vegans or strict vegetarians, who do not consume eggs, dairy, or fish [4].

The third molars are the most impacted teeth, comprising 98% of all impacted teeth, with a prevalence ranging from 18.97% to 30.80%, varying among different ethnic groups and populations [5]. These teeth are often indicated for removal due to inflammatory complications, developmental abnormalities, improper positioning, or caries. Furthermore, their presence may hinder orthodontic treatment and prosthodontics, and may serve as inflammatory foci that impacts the ability to undertake effective treatment of malignant tumors, including the initiation of antiresorptive therapy. Consequently, third molar extraction is one of the most commonly performed procedures in oral surgery [6].

The healing process of the lower third molar socket involves pain sensation, swelling, and trismus as a natural response to C-reactive protein elevation, oxidative stress, and changes in fibrinogen and blood cell count. There are reports suggesting that plant-based diet may influence the levels of the above-mentioned affecting the soft tissue response to surgical procedure [7,8].

However, despite the widespread and long history of following of lacto-ovo vegetarian (LOV) diets and the surgical removal of third molars, studies investigating the impact of vegetarian diets on soft tissue healing in the oral cavity are lacking in the current literature.

This study aims to evaluate the potential influence of an LOV diet on post-operative wound healing following third molar extraction. The null hypothesis states that a lacto-ovo vegetarian diet has no significant effect on wound healing, swelling, pain levels, or analgesic use after third molar extraction compared to a general diet.

## 2. Materials and Methods

This study was designed as a prospective study and was conducted at the Oral Surgery Department of Wroclaw Medical University between 1st September 2023 and 31st August 2024 on patients referring with need of lower third molar operational extraction. The assignment of the medical intervention was not at the discretion of the investigators. The experiment was approved by the Bioethics Committee at the Wrocław Medical University, Mikulicza-Radeckiego St. 41, 50-367 Wrocław (Approval No. KB 205/2023N). All participants provided two forms of written consent: the first for the third molar surgical procedure and the second for participation in this study. This study was conducted in full compliance with the Declaration of Helsinki and adhered to personal data protection regulations (GDPR).

### 2.1. Inclusion and Exclusion Criteria

The inclusion criterion for this study was the necessity of surgical removal of an impacted mandibular third molar. From a total number of 48 assessed patients, 8 did not met the criteria. One of them due to general medical condition, two of them due to lower molar full eruption and no need of operational procedure, and 5 declined to participate in this study. All of them were treated with a standard care and achieved sufficient treatment despite this. Later on, 3 patients in the control group were excluded from this study as they did not appear at the follow-ups, and 3 more patients were enrolled in that group instead. A total of 40 adult patients of both sexes were eventually enrolled into this study and divided into two equal groups. The first group (LOV) consisted of lacto-ovo vegetarians, while the second group (control) included participants following a general, non-restrictive diet. A mandatory requirement for participation was adherence to the selected diet for at least 7 years.

Patients qualified for this study had to be adults and capable of providing informed consent for both the surgical procedure and participation in this study. Assignment to the study group (LOV) was based on patient history, confirming adherence to a lacto-ovo vegetarian diet for a minimum of 7 years. Participants were recruited from individuals reporting to the Oral Surgery Department at the University Dental Clinic (Wrocław) for planned third molar extraction. The recruitment was done by an investigator, who was not involved in surgery (A.B). Further, every participant drafted the name of the oral surgeon (A.P., K.K., M.M., and S.D.) in a block randomization method and was referred to them for the surgical procedure. Each operator shared similar experience and was blinded to the type of diet of the patient.

The exclusion criteria were as follows:

1. Systemic or local diseases that could compromise healing.
2. Smoking.
3. Pregnancy.
4. Breastfeeding.
5. General and local contraindications to surgical procedure.

### 2.2. Protocol of an Experiment

The schedule of visits included the following steps:

#### 2.2.1. Consultation Visit

This included a prequalification of the patient for surgery, as well as clinical and radiological examinations:

- Assessment of tooth position according to Pell and Gregory’s classification [9].
- Baseline measurement of maximum jaw opening (in mm): the distance between the incisal edges of the upper and lower central incisors at their mesial surfaces.
- Assessment of bacterial plaque presence based on the Plaque Index (PI) according to Silness and Löe.
- Swelling measurement using the Laskin method: swelling was assessed based on distances measured at predetermined time points before and after surgery (A, B, C), along with the evaluation of trismus and swelling post-operatively [10].

**Figure 1:**
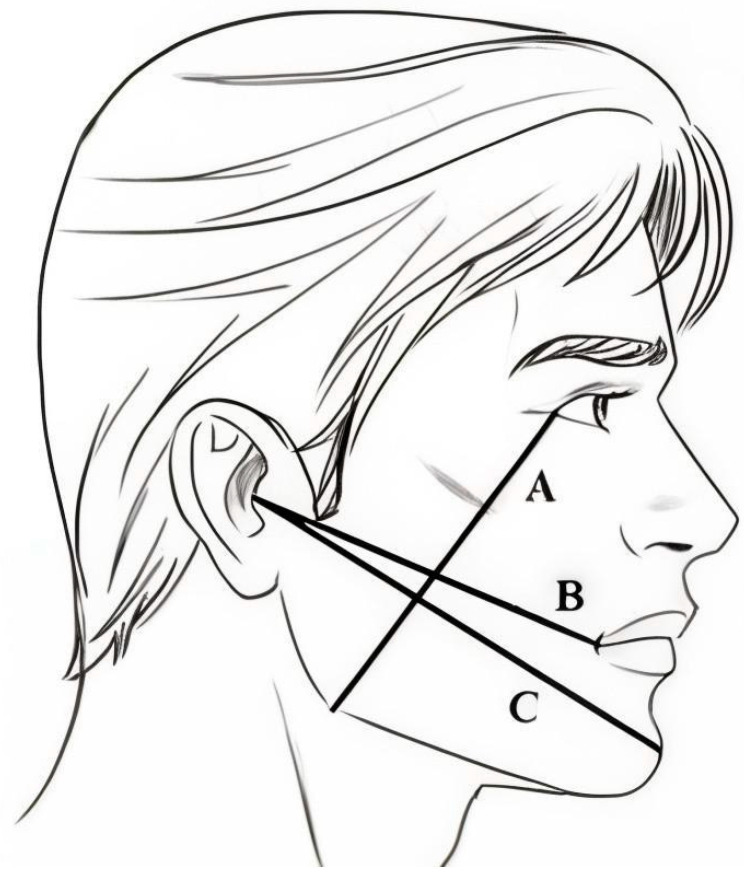
Face lines according to Laskin to determine swelling of the face tissues. A—line connecting the outer corner of the eye to Gonion (Go), B—line connecting the tragus of the ear to Cheilon (Ch), C—line connecting the tragus of the ear to Pogonion (Pg).

The measured distances were as follows:

- A: The vertical distance in millimeters from the lateral palpebral angle to the gonial angle.
- B: The horizontal distance in millimeters from the lower edge of the earlobe to the external angle of the mouth (corner of the mouth).
- C: The horizontal distance in millimeters from the lower edge of the earlobe to the midpoint of the mandibular symphysis (Hirota point) (Figure 1).

#### 2.2.2. Surgery Procedure of the Third Molar Operation and Measurements Following It

All participants enrolled for study were referred to 4 surgeons in a random manner, as each surgeon performed an equal number of 10 operations. For every participant, the surgical procedure was a standard surgical management of the lower third molar. For each surgery, a mucoperiosteal flap was raised under LA using 3.4 mL of 4% articaine with 1:100,000 epinephrine (Septanest 1:100,000, SEPTODONT 58, Saint Maur des Fossés, France). Surgical bur no. 4 was used to perform osteotomy, while surgical bur no. 701 was used to section the tooth. The impacted tooth was then delivered using tooth elevators and dental forceps. Later, the flap was repositioned and stabilized with 5-0 simple interrupted sutures (Seralene^®^, Serag Wiessner, Naila, Germany). Every participant received written instructions regarding the maintenance of local haemostasis, oral hygiene, diet, and prescribed medications. Post-operative medications included oral analgesic and mouthwash and did not include any antibiotic. Patients were asked to rinse with 0.12% chlorhexidine solution twice daily for 7 days, starting the next day after surgery. The only allowed analgesic was oral tablets of paracetamol 500 mg. Patients were instructed to take one tablet as necessary, with a maximum of 6 tablets a day. It was not allowed to add any other analgesic or medication to the prescription. Patients were also asked to note down the amount taken.

Subsequent procedure following parameters were reported:

- The length of soft tissue wound as a distance from the most distal aspect of the 2nd molar crown to the most far aspect of the oral mucosa wound at the edge of the mandible was noted each time following surgery.
- Operational discomfort as VAS (Visual Analogue Score) assessment, using a VAS ruler, with zero representing no pain and 10 the worst pain the patient had ever experienced.
- The level of osteotomy measured in mm from the most coronal aspect of the alveolar process at 2nd molar level to the most apical level of osteotomy.
- The total length of the procedure.

#### 2.2.3. Follow-Up Procedures

The patients were scheduled for follow-up visits on the 3rd and 7th day after surgery. During these appointments, the following parameters were assessed:

- Measurement of maximum jaw opening (mm).
- Measurement of tissue swelling according to the scheme (measurements A, B, C) (mm).
- Measurement of wound length (mm).
- Assessment of bacterial plaque presence based on the Plaque Index (PI) according to Silness and Löe.
- Patient-reported pain over the first 3 days following surgery using the VAS.
- Patient-reported use of painkillers during the first 3 days following surgery, recorded as the number of 500 mg paracetamol tablets taken daily.

On the 7th day post-surgery, the following procedures were performed:

- Suture removal.
- Measurement of maximum jaw opening (mm).
- Measurement of tissue swelling according to the scheme (measurements A, B, C) (mm).
- Measurement of wound length (mm).
- Assessment of bacterial plaque presence based on the Plaque Index (PI) according to Silness and Löe.
- Patient-reported pain during days 3–7 following surgery using VAS.
- Patient-reported use of painkillers during days 3–7 following surgery.
- Assessment of wound closure using the Early Healing Index (EHI) scale.

### 2.3. Statistical Analysis

Sample size was calculated based on confidence level at 95%, margin of error 10%, population proportion at 4% (estimated distribution of LOV diet followers in population). The achieved minimal sample size due to such calculation was 15 participants in each group. We decided to increase the number of participants in each group to 20, as such a sample size of 2 equal groups differing by one variable of total 40 participants has been proposed as sufficient large in the literature before [11]. All data were tested for normality distribution with the Shapiro–Wilk test. In the case of normal distribution, the data were further tested with *t*-test or Welsch’s *t*-test, or ANOVA test in the case of more groups to be tested. In the case of non-normal distribution, a nonparametric Mann–Whitney U test for dependent variances or Wilcoxon test for independent variances were used, or in the case of more groups to be tested, the Friedman test was implemented with significance set at *p* < 0.05 (STATISTICA v. 13.3, TIBCO Software Inc., Palo Alto, CA, USA).

## 3. Results

### 3.1. General Data

The LOV group consisted of significantly more females (95%) and generally younger patients (25.2 (±5.1)) when compared to the control group (31.1 (±9.4)); however, without statistically important differences in that scope (Table 1). A slight majority of the LOV diet followers was taking food supplements: 11 of them vitamins D3 and B12, 10 of them omega-3 acids, and 3 of them iron and magnesium supplements. In control group, the supplementation admitted only two participants: one of them supplemented iron, magnesium, and vitamin D3, while other vitamin D3 and magnesium.

### 3.2. Pre-Operational and Operational Data

The LOV group consisted of lower wisdom teeth, which were generally deeper impacted, as 30% of the cases were the CIII class of impaction and only 15% cases were most superficial IA impaction. In the control group, most IA impaction comprised of 40% of cases and only 15% were deeply impacted IIIC third molars. However, the data did not reach the statistically important differences in that matter (Table 1). In terms of presurgical hygiene indices of study participants, no statistically important differences were found. The initial jaw opening in the LOV group was statistically significantly lower and was also accompanied with statistically significantly smaller face dimension, as all face lines implemented in this study were also smaller (Table 2 and Table 3).

Reasonable to the depth of the impaction, the procedure in the LOV group involved higher levels of osteotomy; however, surprisingly, the total length of the procedure was shorter in that group (Table 2).

### 3.3. Results of Soft Tissue Healing

**Figure 2:**
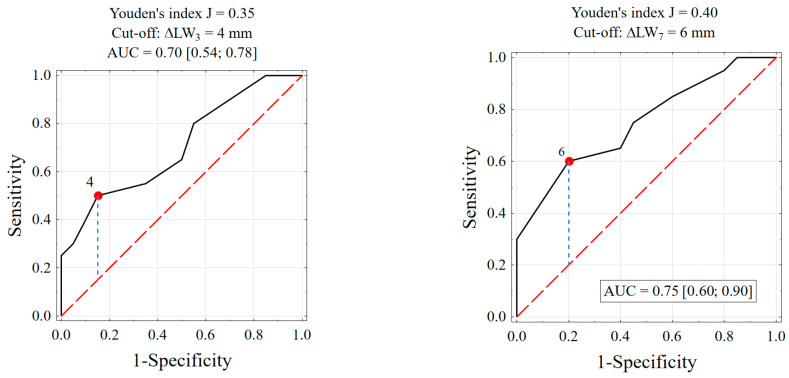
ROC curves to prognose the following of an LOV diet on the base of wound reduction after 3 and 7 days, proposed threshold settings and Area Under the Curve (AUC) at 95% confidence interval.

The initial length of the oral mucosa wound was comparable for both groups (13 vs. 14). It dropped after 3 and 7 days of healing in both groups. However, the reduction of the wound length (ΔLW) was significantly higher in the LOV group. On the contrary, the EHG index did not differ significantly on the 7th day of observation between the groups and was quite comparable (Table 2, Table 3 and Table 4). To judge if the female gender domination in the LOV group influenced the results, a further analysis was made. First, analysis of a receiver operating characteristic (ROC) curve were done. It might be assumed that a “success” of healing is the reduction of wound length at 4 mm or more on the 3rd day and 6 mm or more at the 7th day following surgery, and such thresholds were established (Figure 2). A confounder variable apart from gender might have been patient age and length of the procedure, which are continuous variables. They were transferred to dichotomous variables. Patients were classified based on ROC analysis to one of the following two groups: age below or above 29 years.

- For test “Age ≤ 29 years”, test sensitivity (SENS) was 85.0%, and test specificity (SPEC) was 55.0%.
- For test “Length of the procedure ≤ 22 min.”, SENS was 60.0%, and SPEC was 70.0%.
- For test “Female gender”, SENS was 76.9%, and SENS was 37.5%.

For such thresholds, the logistic regression analyses were caried out separately for the 3rd and 7th days of observation. The results showed significantly important differences only for the LOV diet variable (Table 5 and Table 6). To further evaluate the influence of the LOV diet on wound healing at the end of the observation period (ΔLW_7_ ≥ 6 mm), the four-square contingency table and Fisher’s exact test were applied (Table 7). The probability of wound reduction at 6 mm or larger on 7th day after the procedure for the LOV group was 6 times higher when compared to the control group (*p* = 0.024, OR = 6.00). To exclude the potential influence of gender, age, and length of the procedure on that result, the Mantel–Cochran–Haenszel test was additionally caried out. The final adjusted odds ratio was aOR = 4.52 (*p* = 0.014); hence, it might be assumed that the probability of wound reduction at 6 mm or more on the 7th day after surgery in the LOV group is over 4 times higher when compared to the control group.

**Table 3:** Basic descriptive statistics of parameters in the vegetarian and control groups and results of significance tests.

Parameter	Group		Test Result
	LOV N = 20	Control N = 20	
Jaws opening on the day of surgery (mm)	45.7 (5.0)	48.0 (3.6)	*p* = 0.104 ^d^
On the 3rd day following surgery (mm)	37.1 (9.5)	44.3 (5.1)	***p* = 0.005** ^e^
On the 7th day following surgery (mm)	41.7 (7.3)	44.3 (4.4)	*p* = 0.183 ^e^
Test result:	***p* < 0.001** ^g^	***p* = 0.001** ^g^	
Length of the wound on the day of surgery (mm)	13 [11; 15]	14 [10; 15]	*p* = 0.787 ^f^
Length of the wound on the 3rd day following surgery (mm)	10 [9; 10]	12 [10; 12]	***p* = 0.006** ^f^
Length of the wound on the 7th day following surgery (mm)	8 [6; 8]	10 [8; 11]	***p* = 0.001** ^f^
Test result:	***p* < 0.001** ^h^	***p* < 0.001** ^h^	
Line A on the day of surgery (mm)	95 [90; 100]	98 [95; 100]	*p* = 0.208 ^f^
Line A on the 3rd day following surgery	99 [95; 100]	100 [100; 105]	***p* = 0.042** ^f^
Line A on the 7th day following surgery	95 [92; 100]	100 [98; 106]	***p* = 0.001** ^f^
Test result:	***p* = 0.001** ^h^	***p* < 0.001** ^h^	
Line B on the day of surgery (mm)	106 [100; 110]	110 [105; 112]	*p* = 0.086 ^f^
Line B on the 3rd day following surgery	110 [108; 115]	115 [113; 119]	***p* = 0.025** ^f^
Line B on the 7th day following surgery	109 [102; 112]	115 [110; 118]	***p* < 0.001** ^f^
Test result:	***p* < 0.001** ^h^	***p* < 0.001** ^h^	
Line C on the day of surgery (mm)	140 [134; 140]	145 [140; 149]	***p* = 0.040** ^f^
Line C on the 3rd day following surgery	145 [140; 150]	150 [145; 155]	*p* = 0.110 ^f^
Line C on the 7th day following surgery	140 [136; 145]	150 [143; 153]	***p* = 0.011** ^f^
Test result:	***p* < 0.001** ^h^	***p* < 0.001** ^h^	
Plaque index on the day of surgery (score)	0 [0; 1]	0 [0; 0]	*p* = 0.126 ^f^
on the 3rd day following surgery (score)	0 [0; 1]	1 [0; 1]	*p* = 0.108 ^f^
on the 7th day following surgery (score)	0 [0; 1]	1 [0; 1]	*p* = 0.060 ^f^
Test result:	*p* = 0.407 ^h^	***p* = 0.001** ^h^	
Early Healing Index on the 7th day following surgery	2 [1; 2]	2 [2; 3]	*p* = 0.194 ^f^
VAS during the surgery (score)	2 [0; 2]	0 [0; 1]	***p* = 0.045** ^f^
VAS on the 3rd day following surgery (score)	2 [2; 7]	5 [3; 7]	*p* = 0.457 ^f^
VAS on the 7th day following surgery (score)	2 [0; 5]	6 [3; 7]	***p* = 0.017** ^f^
Test result:	***p* = 0.005** ^h^	***p* < 0.001** ^h^	

Mean (SD) or Me [Q1; Q3], ^d^—*t*-test, ^e^—t-Welch test, ^f^—Mann–Whitney U test, ^g^—ANOVA, ^h^—Friedman test.

**Table 4:** Results of chosen measurements at the 3rd and 7th day of follow-ups.

		Jaw Opening 3	Length of the Wound 3	A3	B3	C3	PI3	VAS3	Analgesics 3	EHI
LOV	Avg.	37.1	9.8	97.9	111.9	144.7	0.4	3.9	1.7	1.8
LOV	Mean	36.5	9.5	98.5	110.0	145.0	0.0	2.0	2.0	2.0
Control	Aver.	44.3	11.4	102.1	116.1	148.4	0.7	4.4	2.1	2.1
Control	Mean	43.5	12.0	100.0	115.0	150.0	1.0	5.0	2.0	2.0
		**Jaw Opening 7**	**Length of the Wound 7**	**A7**	**B7**	**C7**	**PI7**	**VAS7**	**Analgesics 7**	
LOV	Avg.	41.7	7.1	95.6	107.5	139.7	0.4	2.5	1.0	
LOV	Mean	43.5	7.5	95.0	109.0	140.0	0.0	2.0	1.0	
Control	Aver.	44.3	9.7	101.9	114.5	147.2	0.7	4.9	2.0	
Control	Mean	43.5	10.0	100.0	115.0	150.0	1.0	5.5	2.0	

**Table 5:** Results of one or more independent variables logistic regression of 4 mm or more wound reduction on the 3rd day following procedure.

Factors	B	*p*-Value	Beta	*p*-Value	OR [95% CI]
Female gender	0.673	0.387	−1.106	0.380	0.33 [0.03; 4.14]
Age ≤ 29 years	0.829	0.286	0.366	0.705	0.70 [0.20; 10.2]
LOV diet	1.753	**0.030**	2.777	**0.047**	16.1 [1.03; 250]
LoP ≤ 22 min	−0.396	0.568	−1.355	0.126	0.26 [0.04; 1.49]

LOV—lacto-ovo vegetarian diet; LoP—length of the procedure.

**Table 6:** Results of one or more independent variables logistic regression of 6 mm or more wound reduction on the 7th day following procedure.

Factors	B	*p*-Value	Beta	*p*-Value	OR [95% CI]
Female gender	1.130	0.147	−0.865	0.469	0.41 [0.03; 4.87]
Age < 29 years	0.762	0.290	0.045	0.960	1.05 [0.17; 6.44]
LOV diet	1.792	**0.018**	2.061	**0.050**	**7.86 [1.01; 63.6]**
LoP ≤ 22 min	−0.084	0.897	−0.891	0.282	0.41 [0.08; 2.08]

LOV—lacto-ovo vegetarian diet; LoP—length of the procedure.

**Table 7:** Results of Fisher’s exact test in the LOV and control groups for the ΔLW_7_ ≥ 6 mm parameter.

Diet	ΔLW7 ≥ 6 mm	ΔLW7 < 6 mm	*p*-Value	OR [95% CI]	aOR [95% CI]
LOV	12 (75.0%)	8 (33.3%)	**0.024**	**6.00 [1.46; 24.7]**	**4.52 [1.27; 16.1]**
Control	4 (25.0%)	16 (66.7%)		1.00 (ref.)	1.00 (ref.)

OR—raw odds ratio, aOR—odds ratio adjusted for patient age, gender, and length of procedure.

### 3.4. Post-Op Complications

Post-operational complication in terms of pain was higher on the day of surgery in the LOV group (1.8 vs. 0.8), comparable on the 3rd day of follow-up (3.9 vs. 4.4), significantly lower on the 7th day of observation in the control group (2.5 vs. 4.9), and was accompanied with the need for oral analgesics. At the end of the follow-up period, the control group had used twice as much analgesia than the LOV group (1.0 vs. 2.0) (Table 2, Table 3, Table 4 and Table 8).

Moreover, the face swelling was smaller in the LOV group, especially on the 7th day following surgery, as the length of all landmark face lines were shorter both on the 3rd and 7th day, reaching significantly important differences at the end of the observation period. However, the trismus was higher in the LOV group, as it reached statistically important differences on the 3rd day of observation ((37.1 (±9.5) vs. 44.3 (±5.1)) and dropped to non-significant differences at the end of the observation period (41.7 (±7.3) vs. 44.3 (±4.4)) (Table 2, Table 3 and Table 4).

## 4. Discussion

Plant-based dietary patients are at risk of nutritional deficiencies, including proteins, iron, vitamin D, calcium, iodine, omega-3 fatty acids, and vitamin B12, all of which play an essential role at every stage of alveolar socket healing. Specific attention should be given to vitamin B12 and iron deficiencies, as these nutrients are predominantly found in meat and might be impossible to replace with a plant-based diet. Studies have shown that vegetarians tend to have lower levels of these micronutrients, which may increase the risk of developing anemia [12].

Moreover, proteins play a crucial role in all stages of wound healing, beginning with the differentiation, proliferation, and metabolic activation of fibroblasts, which drive collagen production, and culminating in the activation of the immune system through the stimulation of leukocyte, monocyte, lymphocyte, and macrophage differentiation, maturation, and function [13]. In a study on tooth-extracted, malnourished mice fed a low-casein diet, Zhang et al. observed delayed wound healing by day 7. Under malnutrition conditions, they reported decreased mRNA expression of genes associated with regeneration and mesenchymal stem cell accumulation, along with an increase in myeloperoxidase and IL-1β mRNA expression [14].

Furthermore, since the human body cannot synthesize eicosanoids, they must be obtained from external sources, primarily marine life, such as fish rich in these fatty acids. Emerging research highlights the supportive role of omega-3 PUFAs in wound healing processes [15]. Eicosanoids and their derivatives, including prostaglandins, leukotrienes, and thromboxane, play critical roles in various physiological systems and pathological processes. These include initiating or suppressing inflammation, contributing to pain perception, regulating cell growth, controlling blood pressure, and modulating the regional blood flow to tissues [16].

However, surprisingly, in the following report, we observed a positive influence of a lacto-ovo vegetarian diet on recovery after oral cavity surgical procedures, particularly in terms of wound healing and the incidence of surgical complications. This observation may be explained, at least in part, by findings reported by other authors. According to these studies, a vegetarian diet may also exert beneficial effects on wound healing, provided it is well-planned and appropriately supplemented. The potential benefits of an LOV diet primarily stem from its more diverse protein sources, low saturated fats, and an abundance of vegetables and fruits. Additionally, vegetarians are more likely to avoid soda drinks and refined carbohydrates, instead consuming higher amounts of complex carbohydrates and dietary fiber. Conversely, excessive meat consumption has been shown to alter intestinal microbiota, and free fatty acids may trigger systemic inflammation through Toll-like receptor activation and might result in a more severe post-operative course [17,18].

Moreover, the benefits of vegetarian diets in preventing and better management of chronic diseases such as type II diabetes and cardiovascular disease have already been well-documented. Hyperglycemia can further impair wound healing by disrupting granulocyte function and contributing to angiopathy or neuropathy [19,20]. While several studies suggest deficits in bone formation after dental extraction in diabetic patients, not all reports are consistent with this conclusion. Overall, healing following tooth extraction in diabetic patients is generally slower compared to non-diabetic individuals, particularly in the initial stages and in cases of poorly controlled diabetes [21]. In the following report, the predominance of female gender in the LOV group was found. It is in line with sociological studies. There are no detailed studies on the Polish population in that scope. Although, reports made among university students in the USA showed that the percentage who identified as vegan or vegetarian increased from 3.4% in 2008 to 5.8% in 2023, percentages that are similar to estimates of vegetarianism in the general population in the US. Importantly, this increase was limited to women. The percentage of women who were vegetarians increased from 4.3 to 8.7% from 2008 to 2023, whereas the percentage of men who were vegetarians dropped from 2008 (3.2%) to 2023 (2.7%) [22]. Reasonably, the imbalance in gender distribution among groups may affect the results in terms of soft tissue healing. However, the results of statistical analysis in respect to length of the wound reduction after exclusion of the potential influence of female gender predominance allowed for certain conclusions in that matter.

The results of our study show a generally lower pain level and consequently lower amounts of oral analgesics needed during the recovery period following lower wisdom tooth extraction in the LOV group. These results were picked up, although that group consisted of generally more complicated procedures involving more invasive courses of osteotomy procedures, even though the procedure by itself was initially estimated as more painful in the LOV group. Several studies evaluated the role of nutrition in pain management. Most of them were conducted on patients suffering from chronic, potentially progressive diseases, but none of them judged the influence of a plant-based diet on post-operative pain and its management [23]. Kaartinen et al. proved in a prospective study on 18 fibromyalgia patients a significantly reduced pain sensation following vegan diet implementation with the comparison to omnivores. First results were already found in just a 3-months period of observation following the carrying out of a vegan diet [24]. In another uncontrolled study, the plant-based diet was reported to have a beneficial influence on pain management in the case of patients suffering from rheumatoid arthritis [25].

Despite the promising findings of this study, certain limitations should be acknowledged. First, the sample size was relatively small, which may limit the generalizability of the results. Larger, multicenter studies are needed to confirm our findings and explore the long-term effects of plant-based diets on post-extraction healing. Additionally, this study relied on self-reported dietary history to classify participants into the LOV and control groups. While efforts were made to ensure accurate dietary classification, potential recall bias or inaccuracies in dietary reporting cannot be entirely eliminated. Another limitation is the lack of detailed nutritional profiling of participants. While adherence to a vegetarian or general diet was verified, no biochemical assessments of micronutrient levels, such as vitamin B12, iron, or omega-3 fatty acids, were conducted.

Furthermore, our study did not account for other lifestyle factors that may differ between vegetarians and omnivores. Vegetarians often engage in healthier behaviours and place a greater emphasis on overall health and wellness, which could contribute to improved healing outcomes [26].

Another important factor that may have influenced the results is the age difference between the groups. The LOV group in our study generally consisted of younger patients, and the mean surgery time was significantly shorter in this group. It is well-established that younger individuals tend to have a better regenerative capacity, enhanced immune function, and a more robust inflammatory response, all of which contribute to more efficient wound healing. Additionally, shorter surgical time is associated with reduced tissue trauma, lower levels of post-operative inflammation, and a decreased likelihood of complications such as infection or delayed healing [27]. These factors should be taken into consideration when interpreting the findings, as they may partially explain the improved healing observed in the LOV group.

## 5. Conclusions

While our study suggests that a lacto-ovo vegetarian diet may positively influence wound healing and post-operative recovery following third molar extraction, additional research is needed to fully understand the underlying mechanisms. Future studies should incorporate larger sample sizes, more detailed methodology including the evaluation of systematic inflammatory markers, detailed nutritional analyses, and consideration of other lifestyle and demographic variables to provide a more comprehensive understanding of the role of diet in surgical outcomes.

## Figures and Tables

**Table 1:** Descriptive statistics and the normality test to determine distribution for selected variables for the LOV group and control group; SD—standard deviation, *p*—probability value.

Parameter	Group		Test Result
	LOV N = 20	Control N = 20	
Sex			***p* < 0.001** ^a^
Female	19 (95%)	8 (40%)	
Male	1 (5%)	12 (60%)	
Age (years old)			*p* = 0.068 ^b^
Mean (SD)	25.2 (5.1)	31.1 (9.4)	
Me [Q1; Q3]	24 [21; 29]	30 [23; 41]	
Min–Max	21–41	18–41	
Tooth			*p* = 1.000 ^a^
38	8 (40%)	9 (45%)	
48	12 (60%)	11 (55%)	
PELL and GREGORY			*p* = 0.068 ^c^
IA	3 (15%)	8 (40%)	
IB	2 (10%)	4 (20%)	
IC	1 (5%)	0 (0%)	
IIA	2 (10%)	0 (0%)	
IIB	5 (25%)	5 (25%)	
IIC	1 (5%)	0 (0.0%)	
IIIC	6 (30%)	3 (15%)	

^a^—exact Fisher’s test, ^b^—Mann–Whitney U test, ^c^—Chi-squared test.

**Table 2:** Results of the pre- and post-operative measurements.

		Jaw Opening 0	Length of the Wound 0	A0	B0	C0	PI0	VAS0	Osteotomy	Length of the Procedure
LOV	Avg.	45.7	13.7	94.9	105.3	137.2	0.5	1.8	2.5	22.2
LOV	Mean	45.5	13.0	95.0	105.5	140.0	0.0	2.0	3.0	20.0
Control	Aver.	48.0	12.8	97.7	109.5	143.4	0.2	0.8	2.2	26.7
Control	Mean	48.0	13.5	98.0	110.0	145.0	0.0	0.0	2.5	30.0

**Table 8:** Descriptive statistics of parameters on post-op pain management in the LOV and control groups and results of significance tests.

Parameter	Group		Test Result
	LOV N = 20	Control N = 20	
Dose of paracetamol needed by patient on the 3rd day following surgery	2 [1; 2]	2 [2; 3]	*p* = 0.072 ^f^
Dose of paracetamol needed by patient on the 7th day following surgery	1 [0; 2]	2 [1; 3]	***p* = 0.007** ^f^
Test result:	***p* = 0.003** ^i^	*p* = 0.646 ^i^	
Osteotomy applied during surgery (mm)	3.0 [1.5; 3.0]	2.5 [0.8; 3.0]	*p* = 0.735 ^f^
Length of the procedure (min)	20 [15; 30]	30 [20; 30]	*p* = 0.108 ^f^

^f^—Mann–Whitney U test, ^i^—Wilcoxon signed-rank test.

## Data Availability

All data can be provided on request.

## Abbreviations

The following abbreviations are used in this manuscript:

LOV	Lacto-ovo vegetarian
LA	Local anesthesia
VAS	Visual analogue score
ΔLW	Reduction of the wound length
